# Agreement between self-reported asthma symptoms and exhaled nitric oxide levels: impact on inhaled corticosteroid prescribing in general practice. An observational study

**DOI:** 10.1186/s13223-019-0390-x

**Published:** 2019-11-21

**Authors:** Raj Gill, E. Mark Williams

**Affiliations:** 1 Swiss Cottage Surgery, Camden CCG, 2 Winchester Mews, London, NW3 3NP UK; 2 Faculty of Life Sciences and Education, University of South Wales, Pontypridd, UK

**Keywords:** Asthma, Review, Nitric oxide, Asthma Control Test, Inhaled corticosteroids, Prescribing

## Abstract

**Background:**

The National Review of Asthma Deaths UK highlighted that 46% of deaths could be avoided and recommended that all sufferers receive a structured asthma annual review which assess asthma control. In primary care this is commonly achieved using symptom-based questionnaires such as the Asthma Control Test (ACT). A newer method of assessing asthma control is Fractional Exhaled Nitric Oxide (FeNO) testing, which is currently recommended for the diagnosis of asthma, but not for monitoring of asthma control. The study aim was to assess the correlation between self-reported symptoms as measured by the ACT and FeNO testing and the subsequent impact of FeNO testing on prescribing of asthma medication.

**Methods:**

A retrospective review of 65 patients who had received both ACT and FeNO testing as part of their asthma annual review. A spearman correlation was used to estimate the correlation between ACT scores and FENO levels. A χ^2^ test was used to compare prompting frequency of the measures and Kendalls τ statistic was made to estimate their concordance and influence on subsequent ICS medication prescription.

**Results:**

The mean age of the participants was 41 years (4–93 years). There was no statistically significant correlation between ACT and FeNO (ρ = 0.195, p = 0.120). The median FeNO was 26 ppb (range 8–279 ppb), and the ACT score 20 (range 5 to 25 points). Furthermore, FeNO more frequently prompts a change in medication than ACT, 66% versus 42% (p = 0.005). A low concordance between the measures was found (Kendall’s τ statistic − 0.321).

**Conclusion:**

FeNO should be considered for monitoring of control in asthma. To balance the cost of implementing this technology into primary care a risk stratified approach could be applied to testing.

## Background

Asthma is a serious global health problem affecting all age groups. The prevalence is high in the UK with 5.4 million people receiving treatment for asthma, which includes 1.1 million children. The UK has the third highest death rates for asthma in high-income countries worldwide, with 1370 asthma across the UK in 2016 [1]. In the National Review of Asthma Deaths UK (NRAD), which examined all recorded deaths from February 2012–Jan 2013, 276 possible asthma deaths were considered in detail by the confidential enquiry panels. One the key findings was that in both primary and secondary care only 16% of patients were judged to have good care, with 46% of deaths being avoidable [2].

One of the key recommendations of the NRAD report is that all patients receive a structured annual review which assesses asthma control [2]. NICE recommends that asthma control should be monitored at every review, using a validated questionnaire such as the Asthma Control Test (ACT) in adults and with children over 5 alongside spirometry or peak flow variability testing [3]. The ACT contains 5 questions that are related to the frequency of both asthma symptoms and use of reliever medication in the previous 4 weeks. The scores in the ACT range from 5 (worst control) to 25 (complete control). Developmental studies have established the cut off points for controlled asthma (ACT, ≥ 20 points), not well controlled asthma (ACT, ≤ 19 point) and uncontrolled asthma (ACT, ≤ 15 points). Readings between 20 and 25 prompt a clinician to continue the current treatment plan or consider a step down in treatment [4]. Studies suggests that both patients and clinicians overestimate the degree of asthma control—which can result in a failure to make necessary interventions such as medication titration, increasing the chance of disability and death [5]. Despite this fact NICE advises against using FeNO testing to monitor asthma control but suggests FeNO can be considered as an option to support asthma management in people who are symptomatic despite using ICS [3]. This recommendation is largely based on evidence from a meta-analysis conducted by the Cochrane airways group, including 4 moderate to high quality studies in over 700 participants including both adults and children. This examined tailoring treatment with FeNO results vs other methods (primarily clinical symptoms). The study shows that there was an overall reduction in daily dosing of ICS in adults, but not children, and there was no difference between groups in all other outcomes including FEV_1_ and asthma exacerbations [6]. More recent evidence shows more promising results for the use of FeNO in monitoring asthma: a large real-world study in the US of nearly 8000 patients compared the management plans of patients’ who have had both clinical assessment of their asthma control and FeNO levels. The study was conducted by asthma specialists, including allergists and pulmonologists, but not primary care physicians. Clinical assessment was concordant with FeNO testing in only 56% of cases. FeNO testing also resulted in alterations in prescription of ICS in 90% of cases [7].

## Methods

### Study design and study population

A retrospective observational study in a Central London GP Surgery. The Surgery has a total asthma register of 489 adults and children, and 80% of these patients are seen for an annual review each year. The practice has a multiple disciplinary team managing asthma including; Doctors, Nurses, Physician Associates and Clinical Pharmacists. Spirometry is conducted routinely for diagnosis of asthma but is not routinely conducted at each annual review. The existing practice was to conduct a symptom-based assessment of a patient’s asthma control using the ACT. FeNO testing was newly implemented in 2018 to complement the existing methods of diagnosis and monitoring asthma. The study was designed to assess the impact of the early implementation of this new technology in the primary care setting. A review was planned after the first batch of tests (100 tests). When measuring FeNO (NIOX VERO, Aerocrine AB, Solna, Sweden) the NICE guidelines cut-off of 40 ppb (parts per billion) for adults and 35 ppbs for children are used to confirm a diagnosis of asthma [3]. NICE gives no specific guidance on values of FeNO for monitoring of asthma, however GINA indicates that evidence of residual Type 2 airways inflammation is seen with a FeNO of 20 ppb—therefore this cut off is used to prompt a step up or step down in treatment [1].

### Data collection

A fixed data collection template was used to collect participant; Age, Gender, FeNO concentration, ACT score, current ICS dose, change in treatment made. The current dose of ICS was recorded as; ICS only, low dose combination, medium dose combination and high dose combination. Change in treatment was recorded as; no change, increase in ICS or decrease in ICS.

Only retrospective data was used and no patient identifiable data was recorded on the data collection template.

### Statistical analysis

Statistical Package for the Social Sciences (SPSS Version 23, USA) was used for statistical analysis. Statistical significance was assumed if p < 0.05. A Shapiro–Wilk test, established that the data was not normally distributed so a Spearman correlation was used to estimate the direction and strength of the correlation between the two groups. An independent samples *t* test was used for pairwise comparisons.

To estimate the concordance between ACT and FeNO measures in their prompt to make changes in the patient’s medication, the measures were grouped according to following logic: FeNO concentration greater than 20 ppb prompts the clinician to increase ICS dosage; similarly, an ACT value lower than 20 prompts the same. Actual ACT and FeNO measures were merged into two groups depending on whether they prompt an increase or not. The distribution of the derived variables was compared using a Chi square test, and a Kendall’s tau statistic was used to calculate the concordance between the measures. Lastly, the frequency of a medication increase was compared between groups of patients having different current medication level using a Chi square test.

## Results

**Fig. 1:**
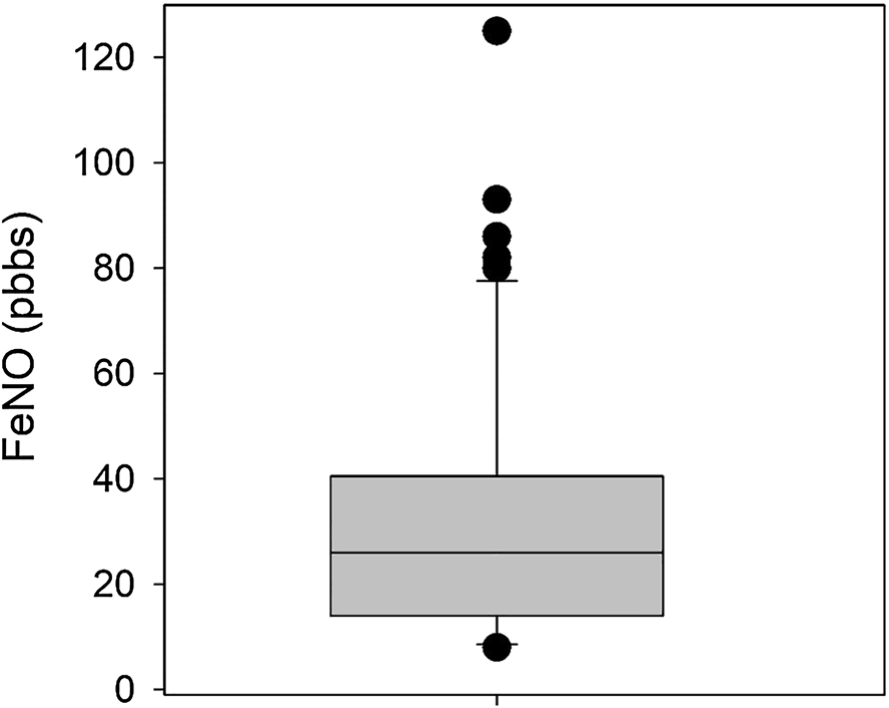
A box-plot of FeNO levels. The median is shown with 25–75th percentile. An outlier of 279 is included in the analysis but not shown

**Fig. 2:**
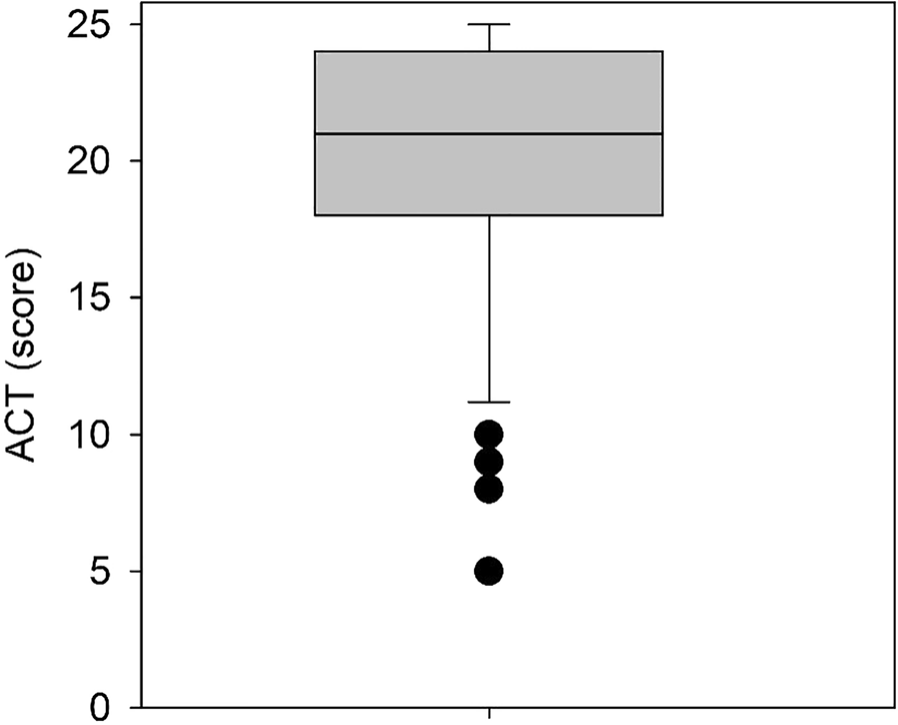
Box plot of ACT scores

A total of 65 patient who had received both the ACT and FeNO as part of their asthma annual review were identified. A retrospective notes review of all these patients was conducted, with the mean age of 41 years (mean ± SD shown, range 4 to 93). Visual inspection of boxplots (see Figs. 1 and 2) revealed that there were several outlying values in both measures (ACT and FeNO) that could also affect the results of correlation analysis if based on the parametric Pearson’s correlation. Therefore, a Spearman correlation was computed which showed a small positive but statically non-significant correlation coefficient (ρ = 0.195, p = 0.120).

Around 29% (19 of 65) of patients were not prescribed ICS, while more than two-thirds of the patients (46 of 65, 71%) were prescribed ICS as part of their treatment plan (Table 1). Of these 46 patients 29% (n = 19) were prescribed a medium or high dose combination, 25% (n = 16) low dose combination and 17% (n = 11) standard ICS dose only. There was only one patient who was currently on a high dose combination inhaler and therefore was added to ‘medium/high dose combination’ group. Over one half of the patients had an increase in dosage after ACT and FeNO tests (n = 36, 55%) and 43% (n = 28) did not change their treatment, with one patient receiving a decrease in the dosage (Table 1).

**Table 1:** Demographic characteristics of the participants

Categorical variables	Count	Percent
Gender		
Male:Female	39:26	60:40
ICS dose		
None	19	29.2
Standard ICS only	11	16.9
Low dose combination	16	24.6
Medium/high dose combination	19	29.2
Change in treatment		
Decrease	1	1.5
No change	28	43.1
Increase	36	55.4

The asthma control level measured by FeNO ranged from 8 to 279 ppb with a median of 26 compared with the mean ± SD, 35 ± 39 (Fig. 1a). The median for the ACT score is 21 compared to the mean of 20 ± 5, range (5–25) (Fig. 1b). There was no correlation between gender and age except in females where there was a positive correlation between ACT and FeNO scores (ρ = 0.539, p = 0.004, n = 26).

To compare the frequency of FeNO and ACT prompting an increase in medication, both measures were merged into two groups according to the rule that a FeNO of 20 ppb and higher prompts an increase in medication and an ACT level lower than 20 prompts the same. FeNO prompts an increase in dosage more often than ACT level (66% n = 43, compared to 42%, n = 27) (p = 0.005, Χ^2^ test), thus FeNO testing has a significant impact on ICS prescribing.

The concordance of these two measures in prompting an increase in medication was estimated by making a cross tabulation of the overall share each combination made (Table 2). In one-third of the cases (n = 21, 32%), both measures showed the same prompts: in 8 cases (12%) both did not prompt an increase and for 13 cases (20%) both measures prompted an increase in medication. Almost half of the cases (n = 30, 46%) should have had an increase in medication based on their FeNO levels, but not based on their ACT level. Vice versa about one-fifth of the sample (n = 14, 21.5%) should had an increase based on their ACT level, but not based on FeNO levels. The concordance between these two measures is negative and low (Kendall’s tau-b correlation: τb = − 0.321, p = 0.010).

**Table 2:** FeNO and ACT concordance in prompting of dosage change

	ACT does not prompt an increase		ACT prompt an increase	
	n	Table %	n	Table %
FeNO does not prompt an increase	8	12.3	14	21.5
FeNO prompt an increase	30	46.2	13	20.0

Changes in treatment by current medication type are shown with a lower proportion of cases receiving an increase dosage in those receiving combination inhalers (p < 0.001) (Table 4).

## Conclusion

The primary study objective was to assess the correlation between patient’s self-reported asthma symptoms as measured by the Asthma Control Test (ACT) and the Fractional Exhaled Nitric Oxide level (FeNO) in a primary care setting. Notes from 65 asthma sufferers were reviewed who had received both FeNO and ACT as part of their asthma annual review. It is assumed that the patient’s self-reported symptoms are an accurate representation of airways inflammation; therefore a high FeNO level should accompany a low ACT score. However, in this study there was no correlation between FeNO and ACT scores (ρ = 0.195, p = 0.120). This data contradicts Stern et al. who retrospectively analysed data from the Childhood Asthma Respiratory Inflammatory Status Monitoring (CHARISM) study, a prospective, open-label, randomized, multicentred, parallel-group study in Italy and The Netherlands [8]. Data was extracted on daily FeNO values and symptom scores over 192 days in 41 children with mild to moderate atopic asthma. The study shows that the majority of subjects had the strongest positive relationship between FeNO values and symptom scores on the same day. Subjects who had severe or moderate exacerbations had a stronger positive cross-correlation between FeNO values and symptom scores, suggesting that concordance of FeNO values and symptom scores is an indicator of increased risk of exacerbation. Stern et al. examined a very specific population in their study, whereas this study covers a much broader age range of patients and therefore a wide range of asthma phenotypes [8]. The concept of concordance between FeNO values and symptom scores has recently been advocated through phenotype-cluster analysis of an asthmatic cohort [9]. It has been found that within a group of adult asthmatic patients, there are subjects in whom FeNO values are strongly associated with asthma symptoms, whereas in others this relationship is weak. This establishes the concept of “concordant” and “discordant” phenotypes of asthma. Cross-correlation analysis of biomarkers and symptoms fluctuating over time might be a new way of quantifying such concordance and might help to identify new “fluctuation phenotypes” of asthma. Crucially Stern et al. suggest that those patients with discordant FeNO values and asthma symptoms may gain the most benefit from more regular FeNO monitoring as it may be the best independent predictor of an exacerbation [8].

The level of association in this study was assessed within the individual demographic groups to ascertain if the association was differently skewed or statistically stronger in the same direction (Table 3). There was only one statistically significant relationship between the two variables and that was a positive correlation between FeNO and ACT amongst females (ρ = 0.539, p = 0.004). This would suggest that there may be a difference in symptom perception in males and females, although this should be judged with caution given the sample size (n = 26).

**Table 3:** Correlation analysis results (overall, by sex and age groups)

	FeNO * ACT correlation	p-value	N
Overall	0.195	0.120	65
Male	− 0.244	0.134	39
Female	0.539^a^	0.004	26
Less than 40 years old	0.037	0.837	33
40 years old and higher	0.282	0.117	32

^a^Marks a significant correlation at 99% confidence interval

A secondary analysis was performed to assess the relationship between FeNO and ACT and a change in medication (only no change and an increase in medication) was considered. The analysis shows that both FeNO and ACT are both statistically significantly higher among patients who had an increase in their dosage, t(62) = − 4.193, p < 0.001 and t(62) = − 2.259, p = 0.027, respectively. When split into two groups according to the following assumption; an ACT level below 20 or a FeNO concentration of 20 prompts a clinician to increase ICS dosage, the analysis shows that FeNO prompted a step up (p = 0.05) in medication more often than ACT did (n = 43, 66% compared to n = 27, 42%). Almost half of the patients (n = 30, 46%) are prompted to have an increase in medication based on their FeNO levels, but not based on their ACT score. Vice versa about one-fifth of the sample (n = 14, 22%) would have an increase in their ICS dose based on their ACT score, but not based on FeNO level. This parallels the findings seen in a secondary/tertiary care setting by Hanania, Massanari and Jain [7]. These authors asked clinicians to rate the level of airways inflammation as low, intermediate or high using clinical measures that were conventionally available to them including: pulmonary function tests, asthma symptoms, control questions and respiratory examinations. Clinicians recorded any changes in medications that would be made according to their clinical assessment, this was then repeated following measurement of FeNO where airways inflammation was recategorized according to the ATS cut-off points for low (< 25 ppb), intermediate (25–50 ppb), and high (> 50 ppb) and clinicians indicated whether their treatment strategy would change armed with this new information. The data show that clinical assessment matched FeNO classification in only slightly more than half the patients (4457 of 7901 56.4%). Subgroup analysis showed that in the high inflammation subgroup (FeNO > 50 ppb), clinician’s assessment matched the FeNO category in only one-third of patients (341 of 1016 33.6%).

When assessing the impact on ICS prescribing; it is seen that FeNO prompted an increase in medication to commence ICS in 100% of patients who were not currently taking an ICS (Table 4). This is consistent with the BTS guidelines which recommend that all patients diagnosed with asthma and those with an intermediate probability of asthma are commenced directly onto a low dose ICS [10]. It also recommends that due to very limited side-effects from long term use of low dose ICS, patients should be maintained on these. This is contrary to NICE which recommends that a SABA may be offered to adults and children newly diagnosed with asthma and may be continued alone in all patients who have infrequent, short-lived wheeze and normal lung function [3].

**Table 4:** Change in treatment by current medication type

	ICS dosage							
	None		ICS only		Low dose combination		Medium/high dose combination	
	n	%	n	%	n	%	n	%
No change in treatment	0	0.0	5	45.5	10	62.5	13	72.2
Increase in dosage	19	100.0	6	54.5	6	37.5	5	27.8

As there was only single case with a decreased medication dosage, it was excluded from the analysis. The difference in the distribution is statistically significant as assessed by Chi square test (χ^2^(3) = 23.006)

The study suggests that FeNO is a superior method of assessing underlying airways inflammation compared to patients self-reported symptoms as measured by the ACT. This substantiates the observation that chronic airways inflammation persists despite lung function tests returning to normal and in the absence of self-perceived symptoms [11]. Guidance which does not support the use of FeNO as a monitoring tool in asthma, has largely considered data which looks at FeNO as a direct reflection of eosinophilic inflammation. Shaw et al. enrolled 118 participations with a primary care diagnosis of asthma and randomised to single-blind trial of ICS based on FeNO measurement or standard step up and down according to BTS guidance [10, 12]. They showed no statistically significant reduction in asthma exacerbations or ICS dosage. Their cut-off for adjustment of therapy was set at 26 ppb for an increase in ICS and 16 ppb or less than 26 ppb on two occasions for a reduction in ICS. These cut-offs were chosen as they were thought to most accurately identify a sputum eosinophil count of greater than 3% or less than 1% respectively, but these cut-offs may not accurately reflect the underlying airway inflammation [13].

### Limitations of study

The sample size was limited to 65, as this study was conducted to capture the impact of FeNO testing following initial implementation. The study, however, covers both genders, a broad age range and the GP surgery is based in a multicultural area with varying socio-economic backgrounds. Another limitation is the use of the ACT in the age range of the population included (4 to 93 years old), the standard ACT was used for all patients, with significant input from parents for younger children. There is however a specific childhood Asthma Control Test (C-ACT) that is validated for use with children 4–11 years old [14]. A standard ACT is used in regular practice at the study site: when children are too young to answer the questions, the questions are answered by the adults accompanying them to the appointment. The accuracy of the result is significantly reduced when the guardian who accompanies the child does not have a detailed knowledge of the child’s asthma. The ACT and c-ACT both suffer from this problem, but this is more pronounced in the ACT as there are no specific questions tailored for younger children to answer. The study does not assess the impact of any adjustments made in therapy and the longer-term impact on asthma care, such as rates of exacerbation; as this was an initial assessment of early implementation of a new technology to the primary care setting. A further prospective study with matched controls following participants over 1 year, would help to elucidate this.

### Clinical application

Within a financially pressurised National Health Service the cost burden of implanting a new technology to monitor asthma must be taken into consideration. FeNO is currently recommended by NICE for the diagnosis of Asthma and suggests that diagnostic hubs could be established in order to achieve economies of scale and improve the practicalities of implementing this recommendation in primary care [3]. The evidence from this study shows that there would also be an increase in use and therefore cost of medication if FeNO was introduced for monitoring of asthma. 100% patients (n = 19) naive to ICS were commenced on treatment and 55% (n = 36) of the total number of patients had a step up in their treatment. Only one patient in this study had a reduction (step-down) in their treatment, a larger study may show the greater impact of stepping down and the costs associated with this. Overall the greatest healthcare costs come from Accident and Emergency (A&E) attendances, hospital inpatient admissions and ITU admissions and these result from acute exacerbations [15]. Understanding whether monitoring asthma using FeNO prevents exacerbations, would help confirm the longer-term cost benefits of FeNO use. The evidence to date on this has been mixed but has been examined in two Cochrane reviews one in adults and one in children. The former evaluated the efficacy of tailoring asthma interventions based on FeNO, in comparison to not using FeNO, that is management based on clinical symptoms (with or without spirometry/peak flow) or asthma guidelines or both, for asthma‐related outcomes in adults [6]. Seven studies with over 1500 participants were included; the FeNO group had a lower rate of exacerbations compared to controls (rate ratio 0.59, 95% CI 0.45 to 0.77), however there was no difference between the groups for exacerbations requiring hospitalisation or rescue oral corticosteroids. For this reason, the authors stated that FeNO could not be recommended universally amongst all adult asthma patients, however the intervention may be useful in those adults with frequent exacerbations. A meta-analysis with the same objective was conducted by the same group in children [16]. The review included nine studies of over 1300 children; there was a significant difference in the number of children having one or more asthma exacerbations over the study period, and this was significantly lower in the FeNO group in comparison to the control group [odds ratio (OR) 0.58, 95% confidence interval (CI) 0.45 to 0.75; 1279 participants; 8 studies]. The number of children in the FeNO group requiring oral corticosteroid courses was lower in comparison to the children in the control group, but there was no statistically significant difference between the groups for exacerbations requiring hospitalisation. The authors concluded again that monitoring with FeNO may be beneficial in a subset of children but could not be recommended universally for monitoring of all children with asthma.

If we consider the model of implementing a diagnostic Hub in primary care (covering a federation of GP surgeries or covering a whole Clinical Commissioning Group (CCG) depending on the geographical size of the CCG) it would be impractical for every patient to receive a FeNO test at their asthma annual review. A referral system would need to be in place and this could risk stratify patients to receive the test. Patients who may be suitable for FeNO monitoring could include: any patients with a confirmed diagnosis of asthma (by FeNO or other means) who are not currently taking an ICS, adults who report symptoms despite being treated with medium/high dose ICS, children who are symptomatic despite treatment with any level of ICS, adults and children with a history of exacerbations. This could have an impact in both adults and children, but the greatest impact may be seen in younger children where peak flow diaries and spirometry are difficult to conduct.

When FeNO was first introduced in the surgery, there was not a strict criteria for use of the test, as the clinical staff become familiar with the test and established the benefit of use. FeNO is currently unfunded by our local CCG and the cost based on the number of tests performed in the surgery is relatively high. The outcome of this study could help to create a selection criterion for patients who should receive the test.

## Supplementary information

**Additional file 1.** Raw data collection FeNO vs ACT.

## Data Availability

Raw data is available in Additional file 1.

## Abbreviations

ACT: Asthma Control Test
CCG: Clinical Commissioning Group
FEV1: Forced Expiratory Volume in 1 s
FeNO: Fractional Exhaled Nitric Oxide
ICS: inhaled corticosteroids
ITU: intensive care unit
NRAD: National Review of Asthma Deaths
